# Editorial: Discovering novel anticancer molecules and revealing the pharmacological mechanism of gastrointestinal carcinoma

**DOI:** 10.3389/fphar.2022.1047705

**Published:** 2022-10-10

**Authors:** Yong Xia, Yan Liu, Young-Do Jung, Sen Lian

**Affiliations:** ^1^ Institute of Precision Medicine, Jining Medical University, Jining, China; ^2^ Department of Urology, New York University School of Medicine, New York, NY, United States; ^3^ Research Institute of Medical Sciences, Chonnam National University Medical School, Gwangju, Korea; ^4^ Department of Biochemistry and Molecular Biology, Southern Medical University, Guangzhou, China

**Keywords:** gastrointestinal carcinoma, anticancer, drug delivery, circRNA, molecular diagnosis, pharmaceutical mechanism

This editorial summarizes the articles published in the special Research Topic of Frontiers in Pharmacology, “*Discovering Novel Anticancer Molecules and Revealing the Pharmacological Mechanism of Gastrointestinal Carcinoma*” aiming to comb out ideas for fighting against gastrointestinal carcinoma. Digestive system carcinoma, including gastric cancer, liver cancer, esophageal cancer, rectal cancer, and colon cancer, constitute the largest group of malignant tumors. Gastrointestinal cancer usually has a poor prognosis because it is generally diagnosed at an advanced stage. Although the implementation of biomarker testing, including HER2, PD-L1, and microsatellite instability (MSI), had positive significance for clinical practice and patient care, there are still many pain points (such as drug resistance, side effects, etc.). Therefore, continuous basic research is of great significance for development of new gastrointestinal cancer treatment strategy. This article will summarize and discuss four perspectives: 1) the molecular diagnosis and prognosis, 2) macromolecular anti-tumor candidates, 3) natural small molecule anti-tumor candidates, and 4) development of novel drug delivery.

The tumor precision diagnosis increasingly relies on biomarkers, which determine the molecular aberrations in tumors for diagnostic, prognostic, or predictive purposes. Many tumor detection markers (including gene mutation, proteins, and RNAs) have been considered as indicators for molecular diagnosis and prognosis. Nowadays, alternative splicing (AS) has become a powerful tool for researchers. Chen et al. integratedly analyzed the relapse-associated AS events and constructed a signature predicting tumor relapse in stage I-III HCC. They established a 16-gene AS signature for predicting tumor relapse in stage I-III HCC and revealed that AS signature was significantly associated with the immune status of the HCC microenvironment (Chen et al.). Besides, nomogram has been used to quantify the likelihood of clinical events and influencing factors and has been widely accepted as a prognosis scoring tool globally in recent years. Chao Zhang et al. developed a novel prognostic nomogram for early gastric cancer (EGC) after surgery to decide whether to use adjuvant chemotherapy (ACT). This nomogram can evaluate the postoperative prognosis of EGC patients: postoperative ACT is recommended for moderate- and high-risk patients but not for low-risk patients (Zhang et al.).

CircRNA is non-coding closed ring RNA with stable structure which does not contain 5′-polarity or 3′- polyadenylated tail (Chen et al., 2021). CircRNAs have been demonstrated to be involved in many digestive tract diseases, including gastrointestinal carcinogenesis and hepatocellular carcinogenesis (Li et al., 2020). Using RNA sequencing, Yuan-yuan Luo et al. found 88 upregulated circRNAs and 72 downregulated circRNAs in human HCC tissues. They discovered that hsa_circ_0098181 was related to the poor prognosis, and increased hsa_circ_0098181 alleviated the malignant phenotype of HCC *via* sponging miR-18a-3p and targeting PPARA (Luo et al.). O-GlcNAc modification has been widely considered as the new hallmark of cancer (Fardini et al., 2013). SLC35B4 has been demonstrated to play an essential role in c-Myc O-GlcNAc modification, which promotes HCC progression. Tao Jiang et al. reported that SLC35B4 in HCC tissues was higher than normal liver tissues, and highly expressed SLC35B4 indicated the poor prognosis for HCC patients (Jiang et al.).

In colorectal cancer treatment, the occurrence of side effects (including nausea, vomiting, oral ulcers, diarrhea, hepatotoxicity, myelosuppression, and immunosuppression) limit the clinical therapies (Leimkühler et al., 2019). Therefore, discovering new medications with high efficacy but low toxicity is needed. β-Elemonic Acid (β-EA) is a natural triterpene derived from *Boswellia* species (Zhang et al., 2019). Researchers found that β-EA inhibited the proliferation and migration of CRC cells *in vitro* and represses subcutaneous tumor-bearing colorectal tumors *in vivo* (Xia et al.). Proteomics and gene ontology (GO) enrichment analysis revealed that the most prominent biological processes mediated by β-EA were mitochondria-related bioprocesses, including translational elongation and termination. Furthermore, β-EA at a low concentration mainly repressed cell cycle by decreasing CDK1, CDK6, and CDC20, whereas high concentration β-EA led to ferroptosis by downregulating ferritin and upregulating ferroxidase, transferrin, and ACSL4 (Figure 1). These findings suggested β-EA could be a potential anti-cancer agent for the treatment of CRC.

**FIGURE 1:**
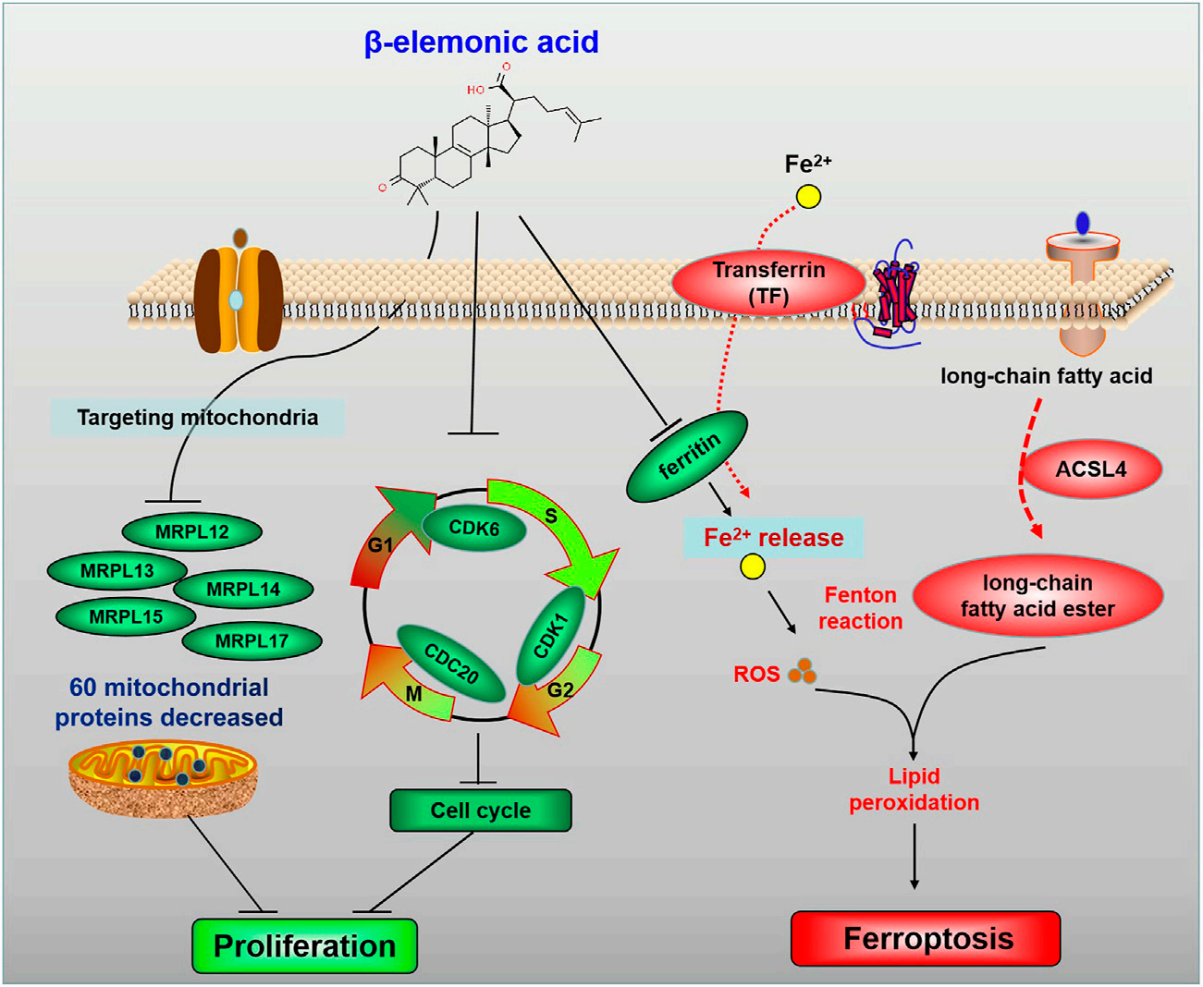
The schematic diagram for the molecular pharmacological mechanism of EA suppressing colorectal cancer (Xia et al.).

The stimuli-responsive polymer-based platform for controlled drug delivery has recently gained increasing attention in treating cancer owing to the fascinating biocompatibility and biodegradability, improved antitumor efficacy, and negligible side effects. Lenvatinib (LEN), a new inhibitor of multiple receptor tyrosine kinases such as VEGFR I-III, FGFR I-IV, PDGFRα and RET, was approved as a preferred drug for the therapy of hepatocellular carcinoma (HCC) (Kudo et al., 2018). But patients who apply LEN suffer side effects, including fatigue, anorexia, diarrhea, weight loss, even nausea and vomiting, or muscle soreness. Nanoparticles exhibit excellent biocompatibility and biodegradability, reduced side effects, prolonged retention time in the blood, and upregulated aggregation in the tumor tissue through the enhanced permeability and retention effect (Feng et al., 2014; Liu et al., 2019). Ding et al. developed methoxy poly (ethylene glycol)—poly (L–phenylalanine–co–L-cystine) (mPEG−P (LP-co-LC)) nanogel loading LEN in the hydrophobic core (NG/LEN) for HCC therapy. The nanogel could achieve concise delivery of LEN *via* the reduction-responsive characteristic of LC. And they found that the intracellular reduction-responsive nanomedicine NG/LEN showed excellent antitumor effect and almost no side effects toward both subcutaneous and orthotopic HCC tumor-allografted mice in comparison to free drug (Ding et al.). The excellent tumor-inhibition efficacy with negligible side effects promotes the potential of NG/LEN for clinical molecular targeted therapy of gastrointestinal carcinoma in the future.

Taken together, in the special Research Topic, the researchers contributed to the gastrointestinal cancer field, including exploring diagnostic and prognostic molecules markers, new anticancer candidates, and drug delivery. We anticipate these findings and research will promote the development of diagnosis and treatment of gastrointestinal cancer.

## References

[B1] Chen L. Wang C. Sun H. Wang J. Liang Y. Wang Y. (2021). The bioinformatics toolbox for circRNA discovery and analysis. Brief. Bioinform 22, 1706–1728. 10.1093/bib/bbaa001 32103237 PMC7986655

[B2] Fardini Y. Dehennaut V. Lefebvre T. Issad T. (2013). O-GlcNAcylation: A new cancer hallmark? Front. Endocrinol. (Lausanne) 4, 99. 10.3389/fendo.2013.00099 23964270 PMC3740238

[B3] Feng S. T. Li J. Luo Y. Yin T. Cai H. Wang Y. (2014). pH-sensitive nanomicelles for controlled and efficient drug delivery to human colorectal carcinoma LoVo cells. Plos One 9, e100732. 10.1371/journal.pone.0100732 24964012 PMC4071001

[B4] Kudo M. Finn R. S. Qin S. Han K. H. Ikeda K. Piscaglia F. (2018). Lenvatinib versus sorafenib in first-line treatment of patients with unresectable hepatocellular carcinoma: A randomised phase 3 non-inferiority trial. Lancet 391, 1163–1173. 10.1016/S0140-6736(18)30207-1 29433850

[B5] Leimkühler M. Hemmer P. H. J. Reyners A. K. L. de Groot D. J. A. van Ginkel R. J. Been L. B. (2019). Neoadjuvant chemotherapy followed by cytoreductive surgery and hyperthermic intraperitoneal chemotherapy for colorectal cancer: A feasibility and safety study. World J. Surg. Oncol. 17 (1), 14. 10.1186/s12957-021-02255-w10.1186/s12957-018-1554-8 30635070 PMC6330449

[B6] Li R. Jiang J. Shi H. Qian H. Zhang X. Xu W. (2020). CircRNA: A rising star in gastric cancer. Cell Mol. Life Sci. 77, 1661–1680. 10.1007/s00018-019-03345-5 31659415 PMC11104848

[B7] Liu L. Dai H. Wu Y. Li B. Yi X. Xu C. (2019). *In vitro* and *in vivo* mechanism of hepatocellular carcinoma inhibition by β-TCP nanoparticles. Int. J. Nanomedicine 14, 3491–3502. 10.2147/IJN.S193192 31190806 PMC6526184

[B8] Zhang Y. Yu Y. L. Tian H. Bai R. Y. Bi Y. N. Yuan X. M. (2019). Evaluation of anti-inflammatory activities of a triterpene β-elemonic Acid in frankincense *in vivo* and *in vitro* . Molecules 24, 1. 10.3390/molecules24061187 PMC6471661 30917586

